# Editorial: Application of genomics and epigenetics in disease and syndrome classification

**DOI:** 10.3389/fgene.2024.1421163

**Published:** 2024-05-01

**Authors:** Yanqi Dang, Wei Wang, Aiping Lyu, Lisheng Wang, Guang Ji

**Affiliations:** ^1^ Institute of Digestive Diseases, China-Canada Center of Research for Digestive Diseases, Longhua Hospital, Shanghai University of Traditional Chinese Medicine, Shanghai, China; ^2^ State Key Laboratory of Integration and Innovation of Classic Formula and Modern Chinese Medicine (Shanghai University of Traditional Chinese Medicine), Shanghai, China; ^3^ Centre for Precision Health, Edith Cowan University, Joondalup, WA, Australia; ^4^ School of Chinese Medicine, Hong Kong Baptist University, Kowloon, Hong Kong SAR, China; ^5^ Department of Biochemistry, Microbiology and Immunology, Faculty of Medicine, University of Ottawa, Ottawa, ON, Canada; ^6^ China-Canada Centre of Research for Digestive Diseases, University of Ottawa, Ottawa, ON, Canada

**Keywords:** disease classification, syndrome classification, genomics, DNA methylation, RNA methylation, protein modification

Genomics and epigenetics have revolutionized disease diagnosis and classification, providing comprehensive insights into disease categorizations (Shen et al., 2018; Koelsche et al., 2021; de Leval et al., 2022). By integrating genomics and epigenetics from the concept of central dogma vs. paracentral dogma (Wang, 2023a; Wang, 2023b), we could account for both genetic predisposition and environmental influences on disease development. In precision medicine, syndrome differentiation plays a crucial role in disease diagnosis and treatment (Dai et al., 2022). Exploring therapeutic strategies to specific syndromes enables more effective disease management. The incorporation of symptomatology and customized prescriptions contributes significantly to the advancement of precision medicine.

We are proud to showcase four featured publications in this Research Topic entitled “Application of Genomics and Epigenetics in Disease and Syndrome Classification”. This editorial aims to provide a concise overview of these articles and highlight their significant contributions to the field.

Genomic studies play a pivotal role in identifying genetic variations associated with diseases. Notably, genome-wide association studies have unveiled single nucleotide polymorphisms (SNPs) associated with specific diseases (Wang et al., 2023). In this Research Topic, Liu et al. found that miR-196a2 rs11614913 and miR-27a rs895819 may influence genetic susceptibility to gastric precancerous lesions (GPL) or gastric cancer (GC). Additionally, they revealed a synergistic effect between miR-196a2 rs11614913 and *Helicobacter pylori* infection in the onset and progression of GPL. A similar study previously indicated that pri-miR-124-1 rs531564 and STAT3 rs1053023 are associated with a higher risk of GC (Mirnoori et al., 2018). These studies demonstrated that SNPs of miRNAs could be significantly associated with GPL or GC. Moreover, Pervin et al. unveiled two novel and clinically impactful metabolic subtypes of pancreatic ductal adenocarcinoma, shedding light on the complexities of substantial intratumoral heterogeneity, consistent with previous studies (Mahajan et al., 2021).

RNA-binding proteins (RBPs) emerge as invaluable biomarkers in disease diagnosis and classification, serving multifaceted roles in detecting disease, monitoring progression, predicting treatment response, and stratifying patients based on molecular profiles. Furthermore, directing interventions towards dysregulated RBPs offers promise for innovative novel treatment strategies across various diseases. Yang et al. showed the pivotal regulatory effect of dysregulated RBPs and their associated alternative splicing events in the development of atopic dermatitis, suggesting promising avenues for therapeutic intervention.

Epigenetic modifications are important in cancer, exerting significant influence in tumor initiation and progression. Profiling these epigenetic alterations helps tumor classification, guiding prognostic assessments and therapeutic choices. Yi et al. made a substantial contribution by summarizing the role of m6A modification in regulating telomerase activity. They comprehensively reviewed the literature, focusing on the applications of the CRISPR system, and the impact of m6A modification on telomerase activity regulation. Additionally, they explored the treatment strategies targeting telomerase activity in age-related conditions and cancer. This review provides the important and the latest information for advancing research in anti-aging therapies and the management of tumor-related diseases.

In summary, genomics and epigenetics play significant roles in disease classification, unveiling the genetic and epigenetic complexity underlying disease progression. Integrating these approaches enhances our understanding of disease mechanisms and facilitates the development of precision medicine strategies. However, the integration of diverse omics data poses challenges due to the complexity and abundance of generated information. Moreover, the utilization of genomic and epigenomic data raises ethical and privacy considerations, emphasizing the need for stringent regulations and guidelines.
